# Further Defining the Phenotypic Spectrum of *B3GAT3* Mutations and Literature Review on Linkeropathy Syndromes

**DOI:** 10.3390/genes10090631

**Published:** 2019-08-21

**Authors:** Marco Ritelli, Valeria Cinquina, Edoardo Giacopuzzi, Marina Venturini, Nicola Chiarelli, Marina Colombi

**Affiliations:** 1 Division of Biology and Genetics, Department of Molecular and Translational Medicine, University of Brescia, 25123 Brescia, Italy; 2 Genetics Unit, IRCCS Istituto Centro San Giovanni di Dio Fatebenefratelli, 25125 Brescia, Italy; 3 Division of Dermatology, Department of Clinical and Experimental Sciences, Spedali Civili University Hospital, 25123 Brescia, Italy

**Keywords:** linkeropathies, *B3GAT3*, Larsen-like syndrome, *B4GALT7*, *B3GALT6*, spondylodysplastic Ehlers-Danlos syndrome, *XYLT1*, *XYLT2*, Desbuquois dysplasia, spondylo-ocular syndrome

## Abstract

The term linkeropathies (LKs) refers to a group of rare heritable connective tissue disorders, characterized by a variable degree of short stature, skeletal dysplasia, joint laxity, cutaneous anomalies, dysmorphism, heart malformation, and developmental delay. The LK genes encode for enzymes that add glycosaminoglycan chains onto proteoglycans via a common tetrasaccharide linker region. Biallelic variants in *XYLT1* and *XYLT2*, encoding xylosyltransferases, are associated with Desbuquois dysplasia type 2 and spondylo-ocular syndrome, respectively. Defects in *B4GALT7* and *B3GALT6*, encoding galactosyltransferases, lead to spondylodysplastic Ehlers-Danlos syndrome (spEDS). Mutations in *B3GAT3*, encoding a glucuronyltransferase, were described in 25 patients from 12 families with variable phenotypes resembling Larsen, Antley-Bixler, Shprintzen-Goldberg, and Geroderma osteodysplastica syndromes. Herein, we report on a 13-year-old girl with a clinical presentation suggestive of spEDS, according to the 2017 EDS nosology, in whom compound heterozygosity for two *B3GAT3* likely pathogenic variants was identified. We review the spectrum of *B3GAT3*-related disorders and provide a comparison of all LK patients reported up to now, highlighting that LKs are a phenotypic continuum bridging EDS and skeletal disorders, hence offering future nosologic perspectives.

## 1. Introduction

Ehlers-Danlos syndrome (EDS) comprises a clinically variable and genetically heterogeneous group of heritable connective tissue disorders (HTCDs) sharing the triad of (generalized) joint hypermobility, cutaneous abnormalities, and internal organ/vascular fragility and dysfunctions. The 2017 EDS nosology recognizes 13 different clinical subtypes and 19 causal genes mainly encoding fibrillar collagens, collagen-modifying proteins, or processing enzymes [1]. A fourteenth subtype has been recently associated with biallelic variants in *AEBP1*, which encodes the aortic carboxypeptidase-like protein (ACLP) associating with collagens in the extracellular matrix (ECM) [2,3,4,5]. In addition to the clinical classification, the 2017 EDS nosology introduced a pathogenetic scheme that regrouped the EDS subtypes into seven functional classes (i.e., disorders) of (a) collagen primary structure and collagen processing, (b) collagen folding and crosslinking, (c) structure and function of the myomatrix, (d) glycosaminoglycan (GAG) biosynthesis, (e) intracellular pathways, (f) the complement pathway, and (g) unresolved forms of EDS. The clinical manifestations of EDS are broad and often overlap other HCTDs including some types of skeletal dysplasias, cutis laxa, hereditary myopathies, and TGFβ-related disorders [6,7]. Hence, intermediate or bridging phenotypes presenting the EDS triad are expected at the boundaries of an evolving nosology.

**Figure 1:**
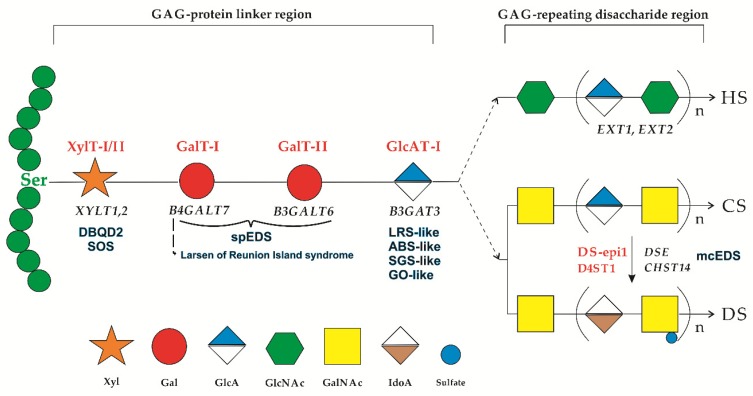
Biosynthetic assembly of the glycosaminoglycan (GAG) backbones of heparan sulfate (HS) and chondroitin sulfate (CS)/dermatan sulfate (DS) chains and related genetic disorders. Each enzyme (in red) and its coding gene (in black) are described near the sugar symbols. After the synthesis of specific core proteins, the synthesis of the GAG-protein linker region is initiated by XylT-I/II, which transfers [Xyl] to the specific Ser residue in the endoplasmic reticulum. The synthesis of the linker region is completed by the consecutive addition of two molecules of [Gal], added by GalT-I/II, followed by the transfer of [GlcA] catalyzed by GlcAT-I in the Golgi. The addition of a [GalNAc] to the linker region commits the growing GAG chain to CS/DS. CS synthesis proceeds with the alternating addition of [GlcA] and [GalNAc] and can be further modified by sulfotransferases. A DS chain is generated after the formation of the chondroitin backbone, when [GlcA] is converted into [IdoA] by DS-epi1, resulting in the formation of the dermatan backbone, where [GalNAc] is sulfated by D4ST1. Alternatively, the addition of the [GlcNAc] to the linker region induces HS biosynthesis. The polymerization of the HS chain is catalyzed by enzymes encoded by the *EXT1* and *EXT2* genes. **Abbreviations**: Ser, Serine; HS, heparin sulfate; CS, chondroitin sulfate; DS, dermatan sulfate; XylT-I/II, xylosyltransferases I/II; GalT-I, galactosyltrasferase I; Galt-II, galactosyltrasferase II; GlcAT-I, glucuronasyltransferase I; DE-epi1; dermatan sulfate epimerases; D4ST1; dermatan 4-*o*-sulfotransferase 1; Xyl, xylose; Gal, galactose, GlcA; glucuronic acid, GlcNAc, *N*-acetylglucosamine; GalNAc, *N*-acetylgalactisamine; IdoA, iduronic acid; DBQD2, Desbuquois dysplasia type 2, SOS, spondylo-ocular syndrome; spEDS, spondylodysplastic EDS; LRS-like, Larsen-like syndrome; ABS-like, Antley-Bixler-like syndrome; SGS-like, Shprintzen-Goldberg-like syndrome; GO-like, Geroderma osteodysplastica-like; mcEDS, musculocontractural EDS.

The multisystemic clinical variability of EDS reflects the numerous functions of collagens and their interactors, among which proteoglycans (PGs) are particularly notable. PGs are structurally complex biomacromolecules that are essential in the development, signaling, and homeostasis of many tissues and organs including bone, cartilage, skeletal muscle, eyes, heart, and skin [8,9,10,11,12,13]. PGs contain one or more variable GAG chains, which are linear polysaccharides consisting of repeating disaccharide blocks attached to a core protein. Depending on the composition of these blocks, the PG superfamily can be subdivided into two major groups: heparan sulfate (HS) and chondroitin sulfate (CS)/dermatan sulfate (DS) PGs. The biosynthesis of GAG chains starts with the formation of a common so-called tetrasaccharide linker region that is covalently attached to a serine residue of the PG core protein (Figure 1). The linker region synthesis is a stepwise process that involves the action of specific glycosyltransferases. It starts with the transfer of a xylose (Xyl) residue by xylosyltransferases I/II (XylT-I/II encoded by *XYLT1* and *XYLT2*, respectively), followed by the addition of two galactose (Gal) residues by galactosyltransferase type I (GalT-I encoded by *B4GALT7*) and type II (GalT-II encoded by *B3GALT6*). The linker region is completed by the transfer of glucuronic acid (GlcA) catalyzed by glucuronosyltransferase I (GlcAT-I encoded by *B3GAT3*), upon which polymerization of the HS or CS/DS chains begins. HS is formed by the alternating addition of disaccharides of N-acetyl-glucosamine (GlcNAc) and GlcA residues, CS by N-acetyl-galactosamine (GalNAc) and GlcA residues, subsequently modified by several sulfotransferases. The formation of DS requires the epimerization of GlcA toward iduronic acid (IdoA), an event accomplished by dermatan sulfate epimerases (DS-epi1 encoded by *DSE*). This allows dermatan 4-o-sulfotransferase 1 (D4ST1 encoded by *CHST14*) to catalyze the 4-o-sulfation of GalNAc, which prevents back-epimerization of the adjacent IdoA (Figure 1) [8,9,10,11,12,13].

The importance of the correct initiation of GAG synthesis is exemplified by the identification of biallelic variants in all genes encoding the key enzymes in the linker region synthesis, leading to a spectrum of severe multisystemic disorders (Figure 1), a.k.a., linkeropathies (LKs) [11,12,13]. 

Two of these LKs, GalT-I- and GalT-II-deficiency, fit with the EDS spectrum and are recognized in the 2017 EDS nosology as spondylodysplastic EDS (spEDS) that also includes patients with mutations in *SLC39A13* encoding the ZIP13 protein involved in the influx of zinc into the cytosol [1,14]. According to the 2017 nosology, spEDS is suggested by two major criteria, short stature and muscle hypotonia, plus characteristic radiographic abnormalities and at least three other minor criteria (general or gene-specific) [1]. At present, 10 patients with molecularly confirmed *B4GALT7*-spEDS have been reported [14,15,16,17,18,19,20,21], 46 with *B3GALT6*-spEDS [14,22,23,24,25,26,27,28], and nine with *SLC39A13*-spEDS [14,29,30,31]. A further 22 patients, all with the same homozygous *B4GALT7* p.(Arg270Cys) missense variant, have been characterized in the ethnic group called white creoles living on Reunion Island (Larsen of Reunion Island syndrome) [32]. 

Molecular defects in *XYLT1* have been associated with Desbuquois dysplasia type 2 (DBQD2) with 28 molecularly proven patients reported hitherto [33,34,35,36,37,38,39,40], while *XYLT2* mutations cause the so-called spondylo-ocular syndrome (SOS), which has been described in 20 patients thus far [41,42,43,44,45,46]. Concerning *B3GAT3*, 25 patients from 12 families have been recognized, but range in severity from mild to severe and resemble Larsen (LR)-, Antley-Bixler (AB)-, Shprintzen-Goldberg (SG)-, and Geroderma osteodysplastica (GO)-like syndromes [26,47,48,49,50,51,52,53]. Further downstream in the GAG biosynthetic pathway, mutations in the *CHST14* and *DSE* genes have been reported in musculocontractural EDS (mcEDS) type 1 and 2, respectively [14].

Herein, we report on a 13-year-old girl ascertained as a suspect of EDS. Trio-based exome sequencing (ES) revealed compound heterozygosity for two likely pathogenic variants in *B3GAT3*. We provide a comparison of the patient’s clinical features with those of the other LK patients reported so far, thus offering future perspectives for clinical research in this field.

## 2. Patient and Methods

### 2.1. Ethical Compliance

The patient was evaluated at the specialized outpatient clinic for the diagnosis of EDS and related connective tissue disorders (i.e., the Ehlers-Danlos Syndrome and Inherited Connective Tissue Disorders Clinic, CESED), at the University Hospital Spedali Civili of Brescia. Molecular analysis was achieved in compliance with Italian legislation on genetic diagnostic tests and the patient’s parents provided written informed consent for the publication of clinical data and photographs according to the Italian bioethics laws. This study followed the Declaration of Helsinki’s principles and was carried out from routine diagnostic activity; a formal ethics review was therefore not requested. 

### 2.2. Amplicon-Based Exome Sequencing

Genomic DNA from the proband and her parents was extracted from peripheral blood leukocytes by standard procedures. Mutational screening was performed by trio-based ES using the Ion Proton platform and the AmpliSeq technology following the manufacturer’s recommendations (Thermo Fisher Scientific, South San Francisco, CA, USA). Briefly, whole exome libraries were prepared using the AmpliSeq Exome RDY kit for library preparation. The template preparation of the libraries was performed using the Ion PI Hi-Q OT2 200 kit on the Ion OneTouch 2 starting from 8 µl of the 100 pM libraries. Template preparation and sequencing runs were performed with the Ion PI Hi-Q Sequencing 200 kit. The templated Ion Sphere Particles (ISP) were enriched for positive ISP using the Ion OneTouch ES and sequenced on the Ion Proton with the Ion PI chip v3. Basecalling and sequence alignment against hg19 genome assembly were performed using Ion Torrent Suite software 5.6, and genetic variants were identified using Torrent Suite Variant Caller pipeline 5.6. Variants were decomposed and normalized using the vt tool [54], filtered for quality using GARFIELD-NGS [55], and annotated using ANNOVAR [56]. Variants were filtered according to the following criteria: (i) MAF < 0.01 in 1000G and ExAc v.0.3 populations; (ii) predicted to alter protein product, namely missense, stop-affecting or splice-affecting variants; and (iii) not present in our internal database. Filtered variants were then prioritized based on DANN [57] and M-CAP [58] scores to retain the most likely deleterious variants. RVIS [59] and GDI [60] scores were used to prioritize more intolerant genes. Finally, we only considered variants with a perfect segregation among the parents and proband according to a recessive/de novo model of transmission. To evaluate the putative pathogenicity of the *B3GAT3* variants, we used the following mutation prediction programs: SIFT [61], Mutation Taster [62], CADD [63], PROVEAN [64], GERP++ [65], UMD_prediction [66], LRT [67], Fathmm-MKL [68], VEST [69], and FitCons [70].

### 2.3. Sanger Sequencing

The *B3GAT3* variants identified by ES (reference sequences: NM_012200.3, NP_036332.2) were confirmed by Sanger sequencing with the BigDye Terminator v1.1 Cycle Sequencing kit on an ABI 3130XL Genetic Analyzer according to the manufacturer’s protocols (Thermo Fisher Scientific, South San Francisco, CA, USA) with specific primer sets amplifying exon 3 and exon 4 of *B3GAT3*, respectively (Supplementary Table S1). The sequences were analyzed with Sequencer 5.0 software (Gene Codes Corporation, Ann Arbor, MI, USA) and variants were annotated according to the Human Genome Variation Society (HGVS) nomenclature by using the Alamut Visual software version 2.11 (Interactive Biosoftware, Rouen, France). 

## 3. Results

### 3.1. Clinical Findings

The proband (LOVD ID #00235371), an Italian 13-year-old girl, was born to non-consanguineous healthy parents and had a healthy sister. Clinical history was remarkable for birth at 41 weeks (height 49 cm, weight 3.2 kg) after induced labor associated with perinatal respiratory distress, anterior ectopic anus, and congenital hip dislocation treated with hip abduction braces, severe neonatal hypotonia, and delayed motor development (delay in walking, first step at three years of age, and acquisition of fine motor skills). She was discharged from the neonatology unit with a diagnosis of generalized joint hypermobility. Medical history further included propensity to develop ecchymoses and surgically treated umbilical hernia. At one year of age, total skeletal x-ray disclosed severe kyphoscoliosis unsuccessfully treated with orthopedic corset, atlantooccipital instability, bilateral radio-ulnar synostosis, abnormalities of the proximal humeral epiphyses, metaphyseal flaring, long and thin bones with widened metaphyses, and delayed bone age. Cervical spine imaging showed significant atlantoaxial and atlanto-occipital instability with flexion and extension. At the age of two, a heart ultrasound revealed an atrial septal defect, which was surgically treated three years later due to tachyarrhythmia and pulmonary hypertension. At ages two and three, respectively, either a clinical diagnosis of SGS or of an unspecified EDS was given. Genetic analyses were not performed. At age four, dual-energy x-ray absorptiometry (DEXA) disclosed severe low bone mineral density for sex and age (z-score < 2 SD) and bisphosphonate treatment was commenced. The progressive kyphoscoliosis and cervical spine instability were surgically treated at age six and 12 without satisfactory improvement. Ophthalmologic evaluation revealed refractive errors such as astigmatism and strabismus. At six years of age, progressive height deficit related to GH deficiency was noticed. GH therapy was started with a discrete outcome, but treatment was interrupted two years later due to the worsening of side effects. Since infancy, the patient suffered from recurrent dislocation of the elbows, shoulders, and knees as well as chronic myalgia of the lower limbs and severe foot pain.

**Figure 2:**
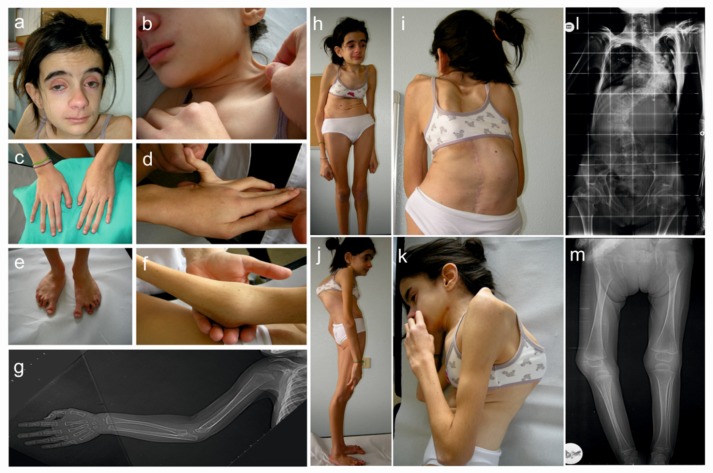
Clinical findings of the patient. Facial dysmorphism: enophthalmos, midface hypoplasia, prominent forehead, micrognathia, low-set ears, and short neck (**a**), skin hyperextensibility over the neck (**b**), long fingers with spatulate distal phalanges and clinodactyly of the fifth finger (**c**), joint laxity of the fifth finger (**d**), foot deformities: sandal gaps, severe pes planovalgus and clinodactyly of the toes (**e**), elbow deformity with reduction in the range of motion (**f**), radioulnar synostosis (**g**), severe kyphoscoliosis (**h**–**l**), pectus carinatum (**h**), muscle hypotonia (**h**–**k**), and metaphyseal flaring (**m**).

On examination, at 13 years of age, she presented with short stature (height 130 cm; genetic target 169 cm, arm span/height ratio, normal value < 1.05), a weight of 25 kg, severe kyphoscoliosis, short neck, pectus carinatum, genua valga, and muscle hypotonia (Figure 2). Facial dysmorphism included enophthalmos, midface hypoplasia, dolichocephaly, prominent forehead, low-set ears, blue sclerae, downslanting palpebral fissures, long philtrum, narrow palate, and micrognathia. Foot deformities such as sandal gaps, severe pes planovalgus, and clinodactyly of the toes were present (Figure 2). The patient also showed skin hyperextensibility over the neck, elbow, forearm and knees, easy bruising, mild atrophic scarring, generalized joint hypermobility with a Beighton score (BS) of 6/9, clinodactyly of the fifth finger, and long fingers with spatulate distal phalanges. Cognitive development and mentation were normal. DEXA confirmed severe osteopenia and delayed bone age despite bisphosphonates treatment; no fractures were reported. Echocardiogram revealed minimal mitral, tricuspid, and aortic valves insufficiency. 

Overall, the patient fulfilled the minimal criteria suggestive for spEDS according to the 2017 EDS nosology [1], since she presented two major (short stature, muscle hypotonia), four general minor (skin hyperextensibility, foot deformity, delayed motor development, osteopenia), and several gene-specific minor criteria either for *B4GALT7*-spEDS or *B3GALT6*-spEDS (craniofacial dysmorphism, characteristic radiographic findings including severe kyphoscoliosis, radioulnar synostosis, metaphyseal flaring, bilateral elbow deformities, peculiar fingers, and gJHM with recurrent dislocations) (Table 1, Supplementary Table S2). Nevertheless, since the patient also presented some peculiar features not previously reported in spEDS patients such as dolichocephaly, atrial septal defect, and anterior ectopic anus, we performed trio-based ES.

### 3.2. Molecular Findings

Summary results of ES are reported in Supplementary Table S3 and Figure S1. After application of the filtering pipeline and prioritization of variants by considering only recessive or de novo variants with perfect segregation among trio members (Supplementary Table S4), six candidate genes were identified (Table 1). Among these, five genes with a de novo variant were excluded, since only *B3GAT3*, associated with multiple joint dislocations, short stature, and craniofacial dysmorphism with or without congenital heart defects (OMIM #245600), was consistent with the patient’s phenotype. 

**Figure 3:**
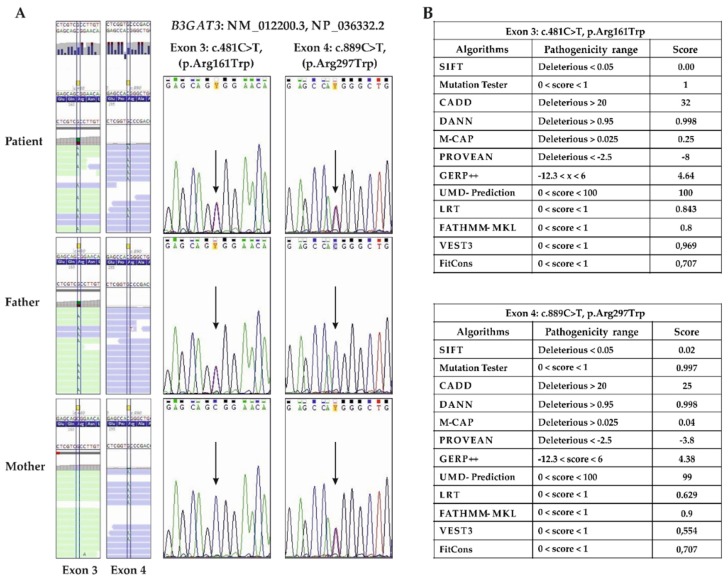
Molecular findings. (**A**) ES data alignments show the compound heterozygosity of the paternal c.481C>T (p.Arg161Trp) and the maternal c.889C>T (p.Arg297Trp) missense variants. Sanger sequencing confirmed the presence of both variants in the proband (arrows). Healthy parents were heterozygous carriers. Mutations are annotated according to HGVS nomenclature (reference sequences: NM_012200.3, NP_036332.2). (**B**) In silico predictions of the pathogenicity of the p.(Arg161Trp) and p.(Arg297Trp) missense substitutions by using 12 different algorithms [61,62,63,64,65,66,67,68,69,70].

**Figure 4:**
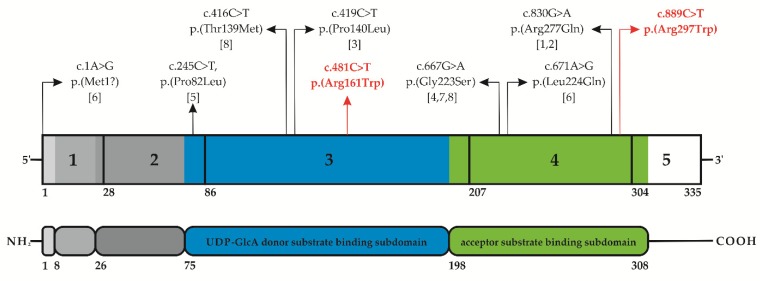
Schematic illustration of the *B3GAT3* structure and glucuronosyltransferase I protein domains. The different *B3GAT3* pathogenic variants found in all patients reported thus far [1-8, present study] are shown over the diagram. The variants identified in this study are in red. Variants are annotated according to HGVS nomenclature (reference sequences: NM_012200.3, NP_036332.2). (**Below**) The protein structure of GlcAT-I is reported with its multiple domains. In different grey scales from the N-terminus (NH_2_) to the C-terminus (COOH), the small cytoplasmic domain (residues 1–7), the transmembrane domain (residues 8–25), and the proline-rich stem region (residues 26–74); in blue and green the catalytic domain consisting of the UDP-GlcUA (uridine diphosphate—(β-D-) glucuronic acid) donor substrate binding subdomain (residues 75–197, in blue), and the acceptor substrate binding subdomain (residues 198–308, in green), according to references [71,72,78].

In particular, the trio analysis revealed the paternally inherited c.481C>T transition in exon 3, leading to the substitution of a highly conserved and positively charged arginine residue with a larger and neutral tryptophan at position 161 [p.(Arg161Trp)] within the donor substrate binding subdomain of the protein [71,72], and the maternal c.889C>T variant in exon 4, which also resulted in the substitution of an arginine residue with a tryptophan [p.(Arg297Trp)], but within the acceptor substrate binding subdomain (Figure 3A and Figure 4) [71,72].

Both variants are annotated in dbSNPs and have extremely low frequencies in population genomic databases. In particular, the paternal variant was observed in three individuals in GnomAD (rs765246909, 3/251290, no homozygotes, total MAF: C = 0.00001194), and the maternal substitution was also observed in three individuals (rs759636773, 3/251080, no homozygotes, total MAF: C = 0.00001195) (queried on 28 May, 2019). Their putative pathogenicity was estimated through 12 different in silico prediction algorithms that agreed to define p.(Arg161Trp) and p.(Arg297Trp) as high impacting variants (Figure 3B).

By using the InterVar (Clinical Interpretation of Genetic Variants) tool [73], both variants were classified as likely pathogenic (class 4) according to the guidelines of the American College of Medical Genetics and Genomics (ACMG) [74] since (i) both variants are missense substitutions in a gene that has a low rate of benign missense variation and where missense variants are a common mechanism of disease (Table 2); (ii) both variants are located in a critical and well-established functional domain of the protein, i.e., the glycosyltransferase domain; (iii) their extremely low frequency in a publicly available population database; (iv) the multiple lines of computational evidence supporting a deleterious effect; and (v) the patient’s phenotype was highly suggestive for a disease with a single genetic etiology. 

The two variants were submitted to the Leiden Open Variation Database (LOVD variants identifiers: #0000480198 and #0000480199). The functional effect of the missense substitutions on reduced/absent enzymatic activity was not verified due to the unavailability of the patient’s fibroblasts.

## 4. Discussion

The umbrella term LK refers to a group of extremely rare and consequently poorly characterized genetic disorders caused by mutations in genes encoding enzymes responsible for the synthesis of GAG side chains of PGs. Nosologic uncertainty characterizes these disorders, thus contributing to the clinical diagnosis of challenging patients, which is not straightforward at all. Indeed, although the linker region is the identical tetrasaccharide sequence for all PGs in all tissues, biallelic variants in the LK genes are associated with apparently different phenotypes that variably affect the skeletal system and skin, even if remarkable similarities between the different LKs are recognizable [8,9,10,11,12,13]. 

In the 2017 EDS nosology, some patients with defects in two out of the five LK genes (i.e., *B4GALT7* and *B3GALT6*) were grouped as spEDS together with those harboring *SLC39A13* mutations, in consideration of the reliable clinical overlap [1], whereas the 22 Larsen of Reunion Island syndrome patients were not included. We have previously suggested that, though some phenotypic variations between Larsen of Reunion Island syndrome and *B4GALT7*-spEDS exist, these conditions should not be considered as different entities [20]. Patients with *B3GAT3*, *XYLT1*, and *XYLT2* mutations were also not classified as spEDS, even though there is a common pathogenic mechanism and numerous shared clinical features. The patient reported in this study corroborates the awareness that LKs are a phenotypic continuum bridging EDS and skeletal disorders. Indeed, the patient was referred to our clinic with a well-founded suspicion of EDS, since she respected the EDS triad and fulfilled the minimal suggestive criteria of spEDS (Supplementary Table S2). In consideration of some peculiar signs not previously associated with spEDS, we performed ES, which revealed compound heterozygosity for two likely pathogenic *B3GAT3* variants.

*B3GAT3* is involved in a spectrum of connective tissue and skeletal disorders. Table 2 summarizes the clinical features of the 26 patients from 13 different families, 11 of which were consanguineous with *B3GAT3* mutations reported so far ([26,47,48,49,50,51,52,53], present study). Among the *B3GAT3*-related disorders, a LRS-like presentation similar to that of our patient was the most common, but more severe phenotypes resembling ABS, SGS, and GO have also been reported (Table 2).

Historically, Baasanjav et al. [47] first described five patients with short stature, radioulnar synostosis, brachycephaly, and cardiac abnormalities. The authors suggested naming this condition as Larsen-like syndrome, *B3GAT3* type. All patients carried the homozygous c.830G>A, p.(Arg277Gln) missense mutation in the acceptor substrate binding subdomain of the protein. Von Oettingen et al. [48] described a 5-year-old boy with a similar phenotype and the same missense variant. Novel findings were developmental delay, refractive errors, pectus carinatum, atlantoaxial and atlanto-occipital instability, and excessive skin wrinkling. Budde et al. [49] reported eight patients from a large consanguineous family with a LRS-like phenotype without cardiac involvement carrying the c.419C>T, p.(Pro140Leu) pathogenic variant in the donor substrate binding subdomain of the protein. Job et al. [51] described the first compound heterozygous patient, a 6-year-old boy, who presented in addition to the typical LRS-like features, hypotonia, hyperextensible skin, and generalized osteoporosis with multiple fractures. The identified mutations were a null allele (c.1A>G, p.Met1?) and the c.671T>A, p.(Leu224Gln) missense substitution in the acceptor substrate binding subdomain of the protein, respectively. Very recently, Colman et al. [53] characterized a 13-year-old girl with a rather mild phenotype with short stature, short neck, craniofacial dysmorphism, joint hypermobility with dislocation, foot deformities, and mild osteopenia without fractures. The patient was homozygous for the c.416C>T, p.(Thr139Met) missense variant in the donor substrate binding subdomain.

Alazami et al. [26] reported a GO–like syndrome in a patient carrying the homozygous c.245C>T, p.(Pro82Leu) missense variant in the donor substrate binding subdomain of the protein. A detailed clinical description is lacking, but short stature, spondyloepimetaphyseal dysplasia, cutis laxa, generalized osteoporosis with fractures, and several bony chondromas were reported.

Finally, Jones [50], Yauy [52], and Colman et al. [53] described the most severely affected patients reported hitherto, all harboring the same c.667G>A, p.(Gly223Ser) missense mutation in the acceptor substrate binding subdomain of the protein. Jones et al. [50] reported a 12-month-old boy with short stature, hypotonia, global developmental delay, radioulnar synostosis, metaphyseal flaring, craniofacial dysmorphism, sandal gap, bilateral club feet, septal defects, and multiple fractures. Novel findings included blue sclerae, bilateral glaucoma, diaphragmatic hernia, small chest, arachnodactyly, lymphedema, hearing loss, and perinatal cerebral infarction. Colman et al. [53] characterized an infant, who died at the age of 2.5 months and showed cutis laxa, contractures of large and small joints, finger and foot anomalies, short neck, severe asymmetric thorax, dolichocephaly and other facial dysmorphic features, multiple long bones fractures, and bilateral corneal clouding. Likewise, Yauy et al. [52] reported six patients, who all died before one year of age, with craniosynostosis, midface hypoplasia, radioulnar synostosis, multiple neonatal fractures, dislocated joints, joint contractures, and cardiovascular abnormalities. The authors suggested that a *B3GAT3*-related disorder with craniosynostosis and bone fragility should be considered as a differential diagnosis in the prenatal period for ABS and in the postnatal period for SGS. Indeed, ABS is suspected during pregnancy if ultrasonography shows craniofacial deformities due to craniosynostosis (a hallmark of ABS), midface hypoplasia, bilateral radiohumeral, or radioulnar synostosis [52,75]. In the postnatal period, SGS can also be suspected, based on the association of craniosynostosis and arachnodactyly. Indeed, SGS is characterized by craniosynostosis and distinctive craniofacial features, skeletal anomalies (arachnodactyly, dolichostenomelia, camptodactyly, pes planus, pectus excavatum or carinatum, kyphoscoliosis), cardiovascular anomalies, intellectual deficiency, and brain anomalies [76].

Interestingly, our patient received a diagnosis of SGS in infancy, though there was the absence of several cardinal features, above all intellectual disability and brain abnormalities. Overall, the clinical presentation of our patient is consistent with a moderate-severe LRS-like phenotype, characterized by short stature, hypotonia, joint hypermobility with recurrent dislocations, radioulnar synostosis, peculiar fingers, foot deformities, midface hypoplasia, short neck, and cardiovascular abnormalities. Of note, although severe osteopenia was present, our patient never experienced fractures. Other features observed in our patient such as skin hyperextensibility, pectus carinatum, atlantoaxial and atlanto-occipital instability, severe kyphoscoliosis, and blue sclerae have been rarely reported in other individuals with *B3GAT3* mutations, while easy bruising, atrophic scarring, and anterior ectopic anus are novel findings (Table 2). Since most of the initial reports focused on a particular aspect of the phenotype, mostly skeletal features, it remains possible that cutaneous and other systemic features that were present have not been described.

The heterogeneous LK phenotypes, ranging from mild to severe and even lethal presentations, seem to be related to specific *B3GAT3* mutations, though there has been a limited number of patients and pathogenic variants described so far (Table 2, Figure 4) [52,53].

In particular, the more severe phenotypes appear to harbor mutations located within the acceptor substrate binding subdomain of the catalytic domain of the protein, whereas more mildly affected patients seem to have mutations in the donor substrate binding subdomain [52,53,77]. The intermediated phenotype of our patient, compound heterozygous for missense variants in both these subdomains (p.(Arg161Trp) in the donor and p.(Arg297Trp) in the acceptor substrate binding subdomain, respectively), corroborates this preliminary genotype–phenotype correlation.

Concerning the p.(Arg161Trp) missense variant, Fondeur-Gelinotte et al. [78] previously demonstrated the importance of this residue in the uridine-diphosphate (UDP)-GlcA donor binding site by site-directed mutagenesis and biochemical analyses. Concerning the consequence of the p.(Arg297Trp) missense variant in the acceptor substrate binding subdomain, in silico modeling using the Hope software [79] suggested that its 3D structure should be severely compromised. Indeed, the wild type residue forms either a hydrogen bond with a glutamic acid at position 295 or a salt bridge with glutamic acid at positions 206 and 295. The differences in size and hydrophobicity between the wild type and mutant residue are predicted to perturb both the hydrogen bond and the ionic interaction, thus likely abolishing the GlcAT-I activity.

The impression that LKs, even though likely not fully characterized yet, should be considered as a phenotypic continuum emerges from the comparison of all LK patients reported up to now (Table 3), which shows several overlapping clinical features, but also some differences. These data support the idea that the enzymes involved in the biosynthesis of the PG linker region may be part of a larger enzyme complex rather than functioning independently, and that the existing phenotypic disparity could be due to varying levels and spatiotemporal gene expression in different tissues [23,48].

The phenotypic features with a high rate of incidence shared among all LKs include short stature, joint laxity with dislocations, craniofacial dysmorphism (especially prominent forehead/eyes and blue sclerae), pectus abnormalities, peculiar fingers, foot deformities, and to a variable degree hypotonia and developmental delay. Delayed cognitive development is instead more frequently observed in *XYLT1*-related DBQD2, followed by *XYLT2*-related SOS, and *B4GALT7*- and *B3GALT6*-spEDS (Table 3).

While joint hypermobility is a common trait of all LKs, joint contractures are more frequently observed in patients with *B3GAT3* and *B3GALT6* mutations. Low bone mineral density/osteopenia and radiographic abnormalities are also very common, whereas generalized osteoporosis and multiple fractures are more frequently encountered in *B3GALT6*-spEDS and in SOS. Likewise, among the numerous radiographic findings, severe progressive kyphoscoliosis is particularly frequent in *B3GALT6*-spEDS, SOS, and DBQD2. In addition to progressive (kypho)scoliosis, short long bones with a monkey wrench or Swedish key appearance of the femora, and advanced carpal and tarsal ossification are also more frequently observed in DBQD2 (Table 3). Of note, the presence or absence of specific hand anomalies, comprising an extra-ossification center distal to the second metacarpal, delta phalanx, or bifid distal thumb phalanx, together with dislocations of the interphalangeal joints are considered radiographic hallmarks distinguishing DBDQ1 (resulting from pathogenic variants in *CANT1)* from DBDQ2 [34]. Platyspondyly, a hallmark of “spondylo”-dysplasia, has never been recognized in *B4GALT7*-spEDS and *B3GAT3*-related disorders, except in the patient reported by Alazami et al. [26], whereas it is frequently reported either in *B3GALT6*- and *SLC39A13*-spEDS or in SOS, whereas in DBDQ2 it was recognized in five out of the nine investigated patients. Radioulnar synostosis seems more specific for *B3GAT3*-and *B4GALT7*-related disorders, the latter including either *B4GALT7*-spEDS (8/10) or Larsen of Reunion Island syndrome (10/21). Radiographic anomalies that are more common in *B3GALT6*-spEDS when compared to other LKs comprise metaphyseal flaring, iliac abnormalities, radial head subluxation/dislocation, which is also frequent in *B4GALT7*-spEDS, and bowing of limbs, which is shared with *SLC39A13*-spEDS (Table 3).

Among the plethora of craniofacial dysmorphism, midface hypoplasia, craniosynostosis, and short/webbed neck are particularly frequent in *B3GAT3*-related disorders. Wide forehead, hypertelorism, and microstomia are more common in *B4GALT7*-spEDS, whereas frontal bossing, micrognathia, and abnormal dentition characterize *B3GALT6*- and *SLC39A13*-spEDS. Flat face is shared among all spEDS subtypes and DBDQ2 (Table 3).

Ocular involvement might facilitate the differential between SOS and other LKs. In particular, the presence of cataract, retinal detachment has been described thus far only in patients with *XYLT2* mutations. Refractive errors/hypermetropia is also frequent in SOS, but has also been found in *B4GALT7*-spEDS and *B3GAT3*-disorders. Apart from eye involvement, deafness is also more frequent in SOS when compared to the other LKs (Table 3). 

Marked cutaneous anomalies including hyperextensible, soft, doughy, thin and translucent skin, and atrophic scarring seem to distinguish spEDS patients, party justifying their inclusion within the EDS spectrum. On the other hand, in all the other LKs, several patients (including ours) with skin hyperextensibility and other abnormalities (including even cutis laxa-like features) have been published (Table 3). As above-mentioned for *B3GAT3*, it remains possible that in more than a few patients, the cutaneous involvement was not investigated. Therefore, further reports are needed to define the real incidence of skin abnormalities in LKs. 

Concerning cardiovascular involvement, anomalies such as septal defects, aortic valve dysplasia, aortic root and ascending aorta dilatation, and mitral valve prolapse, are more recurrent in *B3GAT3*-related disorders as well as in SOS (Table 3). 

## 5. Conclusions

In summary, our findings expand the *B3GAT3* allelic repertoire, corroborate the emergent genotype-phenotype correlations, and confirm the extended phenotypic range of *B3GAT3* mutations overlapping skeletal dysplasia and soft HCTDs including EDS. Furthermore, we provided a comprehensive overview of the phenotypic features of *B3GAT3*-related disorders and of all the LKs, thus offering future nosologic perspectives for either EDS or skeletal dysplasias. Given the convergent pathogenic pathway and the important clinical overlap not only among *B3GAT3*-associated diseases and spEDS (a term that, in our opinion, is unfortunate, since platyspondyly is not present in all subtypes), but also with DBDQ2 and SOS, this group of HCTDs should be considered as a phenotypic continuum and not as distinct entities.. In the genetic era of the classification of disorders, we propose that the term LK is preceded by the specific causal gene. Further reports on additional patients are awaited as well as functional studies on the spatiotemporal expression of the different glycosyltransferases to unravel the molecular mechanisms involved in the pathophysiology of LKs needed to identify potential therapeutic options.

## Figures and Tables

**Table 1:** Candidate genes after selecting variants.

Gene	Variant	Effect	Inheritance Model	Genotype	Clinvar Phenotype
*B3GAT3* (NM_00122200)	c.481C>T (father) c.889C>T (mother)	p.(Arg161Trp) p.(Arg297Trp)	Recessive (comp het)	comp het	Multiple joint dislocations, short stature, craniofacial dysmorphism, and congenital heart defects
*BMP8A* (NM_181809)	c.333G>T	p.(Met111Ile)	Dominant (de novo)	het	
*FES* (NM_0002005.3)	c.1778G>A	p.(Arg593Gln)	Dominant (de novo)	het	
*NR2F6* (NM_005234)	c.806C>T	p.(Pro269Leu)	Dominant (de novo)	het	
*PAK2* (NM_002577)	c.303G>C	p.(Gln101His)	Dominant (de novo)	het	
*TRAK1* (NM_001042646)	c.1327G>A	p.(Ser443Gly)	Dominant (de novo)	het	

**Table 2:** Summary of clinical features of all patients with *B3GAT3* variants.

References	Present Patient	[1,2]	[3]	[6]	[8]	[5]	[4,7,8]
Number of patients	n = 1	n = 6	n = 8	n = 1	n=1	n = 1	n = 8
Phenotype	LRS-like	LRS-like	LRS-like	LRS-like	LRS-like	GO-like	ABS/SGS-like
Consanguinity	-	+	+	-	+	+	+
*B3GAT3* variant(s) (NM_012200.3)	c.481C>T c.889C>T	c.830G>A homozygous	c.419C>T homozygous	c.1A>G c.671T>A	c.416C>T homozygous	c.245C>T homozygous	c.667G>A homozygous
Protein Change (NP_036332.2)	p.(Arg161Trp) p.(Arg297Trp)	p.(Arg277Gln)	p.(Pro140Leu)	p.(Met1?) p.(Leu224Gln)	p.(Thr139Met)	p.(Pro82Leu)	p.(Gly223Ser)
**Skeletal**							
**Short stature**	+	+	+	-	+	+	2/5
**Joint hypermobility**	+	+	-	+	+	na	1/2
**Joint dislocations**	+	+	+	+	+	+	3/7
**Elbow joint abnormalities**	+	+	4/8	na	-	na	+
**Multiple fractures**	-	-	na	+	-	+	6/8
**Kyphoscoliosis**	+	-	4/8	+	-	na	1/7
**Scoliosis/kyphosis**	+	0/1	4/8	+	-	+	2/7
**Platyspondyly**	-	-	0/2	-	-	+	-
**Peculiar fingers** (long, slender, tapered, broad, thin, arachnodactyly)	+	+	+	+	+	na	+
**Pectus abnormality**	+	1/6	-	-	-	na	1/2
**Radioulnar synostosis**	+	1/1	2/2	-	-	na	7/7
**Bowing of limbs**	-	na	4/8	+	-	na	0/8
**Metaphyseal flaring**	+	1/1	na	na	-	na	1/2
**Iliac abnormalities**	-	1/1	na	na	na	na	1/2
**Radial head subluxation or dislocation**	-	na	2/2	na	+	na	0/1
**Foot deformity**	+	+	6/8	-	+	na	+
pes planus	+	1/1	na	-	+	na	na
hallux valgus	+	1/1	6/8	-	+	na	na
club feet	-	0/1	na	-	-	na	+
sandal gap between toes	+	1/1	3/8	-	-	na	1/1
**Osteopenia**	+	5/6	na	+	+	+	2/2
**Cervical spine instability**	+	1/1	na	na	na	na	na
**Craniofacial**							
**Midface hypoplasia**	+	+	+	+	+	na	7/7
**Flat face**	-	1/1	na	-	-	na	na
**Craniosynostosis**	+	+	na	+	na	na	4/7
**Frontal bossing**	+	1/6	na	+	-	na	3/7
**Wide forehead**	-	1/1	na	na	-	na	2/2
**Blue sclerae**	+	0/1	-	+	+	na	5/5
**Proptosis or prominent eyes**	-	+	na	-	+	na	5/5
**Downslanting palpebral fissures**	+	3/5	na	+	+	na	1/2
**Low-set ears**	+	2/5	na	na	na	na	1/1
**Depressed nasal bridge**	-	5/6	4/8	-	-	na	4/7
**Small mouth/microstomia**	-	4/6	3/8	-	+	na	2/2
**Long upper lip/long philtrum**	-/+	na	na	na	+	na	2/2
**Cleft palate/bifid uvula**	+/-	na	na	+	+	na	1/1
**Micrognathia**	+	4/6	4/8	na	-	na	0/1
**Short and/or webbed neck**	+	+	2/8	+	+	na	2/2
**Cutaneous**							
**Skin** (hyperextensibility; soft, doughy, thin, translucent skin)	+ (mild)	1/1 skin wrinkling	-	+	-	Cutis laxa	1/2 Cutis laxa
**Easy bruising**	+	0/1	na	na	na	na	na
**Atrophic scarring**	+ (mild)	0/1	na	na	na	na	0/1
**Other**							
**Cardiovascular abnormalities**	+	6/6	0/3	+	na	+	4/8
**Muscle hypotonia**	+	0/1	na	+	na	na	4/4
**Refractive errors/hypermetropia**	+	1/1	na	+	-	na	0/1
**Delayed motor development**	+	1/1	na	+	+	na	1/1
**Delayed cognitive development**	-	-	-	-	-	na	1/1
**Bone chondromas**	-	-	-	-	-	+	-
**Anterior ectopic anus**	+	-	-	-	-	-	-

Note: +, present in all patients; -, absent in all patients; na, not available; ABS, Antley-Bixler syndrome; GO, Geroderma osteodysplastica; SGS, Shprintzen-Goldberg syndrome.

**Table 3:** Clinical features of linkeropathies.

Genes	*B3GAT3*	*B4GALT7*	*B3GALT6*	*SLC39A13*	*XYLT1*	*XYLT2*
**Number of patients**	n = 26	n = 32	n = 46	n = 9	n = 28	n = 20
**Skeletal**						
**Short stature**	18/23	29/29	36/46	+	+	7/17
**Joint hypermobility**	10/19	+	37/46	+	13/14	2/5
**Joint dislocation**	9/25	+	37/46	+	14/14	na
**Joint contractures (hands, elbow)**	19/24	4/9	30/46	3/9	4/5	na
**Low bone density/osteopenia**	10/11	6/32	20/46	7/9	2/2	15/15
**Multiple fractures**	8/18	1/10	21/46	na	na	19/19
**Kypho/scoliosis**	8/24	7/32	32/46	1/7	10/12	12/13
**Platyspondyly**	1/20	-	13/36	+	5/9	18/18
**Peculiar fingers ^a^**	25/25	1/30	13/36	7/7	19/20	9/12
**Foot deformity ^b^**	22/25	9/10	24/46	8/8	9/13	12/12
**Pectus excavatum/carinatum**	3/19	1/1	2/10	na	10/12	4/4
**Radioulnar synostosis**	11/13	18/31	1/36	-	na	na
**Metaphyseal flaring**	3/5	4/8	23/46	4/6	4/4	0/1
**Monkey-wrench femora**	0/4	1/2	na	na	11/12	na
**Iliac abnormalities**	2/4	-	27/46	-	4/4	2/2
**Radial head subluxation or dislocation**	3/5	17/31	15/36	3/6	1/1	na
**Bowing of limbs**	5/19	7/32	13/46	8/8	5/5	na
**Advance bone age/carpal ossification**	0/10	1/32	5/16	na	13/14	-
**Craniofacial**						
**Midface hypoplasia**	24/25	na	8/10	0/6	1/1	na
**Flat face**	1/4	29/32	22/36	1/1	18/18	na
**Craniosynostosis**	12/15	6/8	1/36	-	na	na
**Frontal bossing**	4/12	-	29/46	+	na	na
**Wide forehead**	2/5	29/32	na	na	1/1	1/1
**Blue sclerae**	8/17	6/10	30/46	+	5/6	2/6
**Proptosis or prominent eyes**	12/14	28/30	20/46	+	6/7	na
**Wide-spaced eyes**	3/7	28/32	-	1/1	1/1	4/4
**Low-set ears**	2/8	7/10	20/46	na	na	4/4
**Depressed nasal bridge**	13/20	-	10/46	1/1	21/21	4/4
**Small mouth/microstomia**	10/19	28/30	-	-	2/2	na
**Long upper lip/long philtrum**	4/4	-	15/36	na	6/6	1/1
**Cleft palate/bifid uvula**	4/4	4/31	6/46	3/8	7/16	na
**Micrognathia**	9/17	3/32	14/46	-	3/3	na
**Short and/or webbed neck**	13/19	-	-	8/8	5/5	4/4
**Abnormal dentition**	1/2	6/10	17/46	8/9	3/5	2/14
**Ocular**						
**Refractive errors/hypermetropia**	3/5	12/29	1/10	1/9	1/5	14/14
**Clouded cornea**	2/4	1/30	1/46	-	0/5	na
**Cataract**	0/26	0/30	na	na	0/5	18/20
**Retinal detachment**	0/26	Na	na	na	0/5	9/16
**Cutaneous**						
**Hyperextensible, soft, doughy, thin, translucent skin; cutis laxa**	4/15	30/32	29/46	+	2/2	1/5
**Atrophic scarring**	1/5	4/32	10/46	5/7	na	na
**Other**						
**Cardiovascular abnormalities**	13/20	-	2/36	-	1/9	7/19
**Muscle hypotonia**	6/7	10/32	21/46	3/3	2/2	10/10
**Delayed motor development**	5/5	8/10	12/46	3/6	10/13	6/13
**Delayed cognitive development**	1/18	19/32	14/46	0/6	17/19	9/17
**Deafness**	1/3	2/32	2/46	-	2/8	12/20

Note: na, not available; +, present in all patients; -, absent in all patients; ^a^: Peculiar fingers including long, slender, tapered, broad, thin, arachnodactyly; ^b^: Foot deformity including pes planus, hallux valgus, club feet, sandal gap; *B3GAT3*: ([26,47,48,49,50,51,52,53], present patient); *B4GALT7*: [15,16,17,18,19,20,21,32]; *B3GALT6*: [22,23,24,25,26,27,28], *SLC39A13*: [29,30,31], *XYLT1*: [33,34,35,36,37,38,39,40]; *XYLT2*: [41,42,43,44,45,46].

## Supplementary Materials

The following are available online at https://www.mdpi.com/2073-4425/10/9/631/s1, Table S1. Primers. Table S2. Clinical features of the present patient and comparison with spEDS and *B4GALT7*-LRS. Table S3. Summary of exome sequencing results. Figure S1. Coverage distribution. Table S4. Variants with perfect segregation among trio members.
