# Supplementary material for: Further Defining the Phenotypic Spectrum of B3GAT3 Mutations and Literature Review on Linkeropathy Syndromes
Source: Genes (Basel). 2019 Aug 21;10(9):631. doi: 10.3390/genes10090631 (PMC6770791; doi:10.3390/genes10090631)
Supplement: Supplementary file 1 [file genes-10-00631-s001.pdf]

Supplementary Table 1.

Primers *B3GAT3* (NM_012200.3)		
**Exon 3**	For3	gcatgaagttgacaggcaag
	Rev3	ccaacggcaacactgctaac
**Exon 4**	For4	gttagcagtgttgccgttgg
	Rev4	tcagcaaggctgatcagaga

Supplementary Table 2

Clinical features of the present patient and comparison with spEDS and *B4GALT7*-LRS		Present Patient	*B4GALT7*-spEDS	*B4GALT7*-LRS	*B3GALT6*-spEDS	*SLC39A13*-spEDS
Major criteria						
	Short stature	+	+	19/19	36/46	9/9
	Muscle hypotonia	+	+	na	21/46	3/3
	Bowing of limbs	-	7/10	-	13/46	8/8
Minor criteria						
	Skin hyperextensibility, soft, doughy, thin, translucent skin	+	9/10	21/22	29/46	9/9
	Foot deformity	+	9/10	na	24/46	8/8
	Delayed motor development	+	9/10	na	12/46	3/6
	Low bone density	+	6/10	-	20/46	7/9
	Delayed cognitive development	-	7/10	12/22	14/46	0/6
Gene-specific minor criteria						
***B4GALT7*-spEDS**	**Bilateral elbow contractures or limited elbow movement**	+	4/9	na	na	na
	**Generalized Joint hypermobility**	+	+	na	37/46	9/9
	**Single transverse palmar crease**	-	6/8	na	-	-
	**Characteristic craniofacial features**					
	Triangular face	-	6/8	na	na	na
	Wide-spaced eyes	-	6/9	22/22	-	1/1
	Proptosis	-	6/8	22/22	20/36	6/8
	Narrow mouth	-	6/8	22/22	-	-
	Low-set ears	+	7/10	na	20/46	-
	Sparse scalp hair	-	5/8	na	4/36	-
	Abnormal dentition	-	5/10	na	17/46	8/9
	Flat face	-	7/10	22/22	20/36	1/1
	Wide forehead	+	7/10	22/22	na	na
	Blue sclerae	+	6/10	na	30/46	9/9
	Cleft palate/bifid uvula	+	3/9	1/22	6/46	3/8
	**Characteristic radiographic findings**					
	**Radioulnar synostosis**	+	8/10	10/21	1/36	-
	Metaphyseal flaring	+	4/8	na	23/46	-
	Osteopenia	+	6/10	-	20/46	7/9
	Radial head subluxation or dislocation	+	5/10	12/21	15/36	3/6
	Short clavicles with broad medial ends	-	3/7	na	-	-
	**Severe hypermetropia**	-	3/10	-	-	1/9
	**Clouded cornea**	-	1/8	-	1/46	-
***B3GALT6*-spEDS**	**Kyphoscoliosis** (congenital or early onset)	+	1/10	-	42/46	0/7
	**Joint hypermobility** (generalized or restricted to distal joint, with joint dislocations)	+	+	22/22 Joint dislocations	37/46	9/9
	**Joint contractures** (congenital or progressive, especially hands)	-	3/7	-	30/46	2/8
	**Peculiar fingers** (slender, tapered, arachnodactyly, spatulated, with broad distal phalanges)	+	3/10	-	13/36	7/7 (tapered)
	**Talipes equinovarus**	-	1/7	-	21/36	-
	**Characteristic craniofacial features**					
	Midface hypoplasia	+	na	na	8/10	-
	Frontal bossing	+	-	-	29/46	9/9
	Proptosis or prominent eyes	-	6/8	22/22	20/46	9/9

Table. (continued)						
		**Present Patient**	***B4GALT7*-spEDS**	***B4GALT7*-LRS**	***B3GALT6*-spEDS**	***SLC39A13*-spEDS**
***B3GALT6*-spEDS**	Blue sclerae	+	6/10	na	30/46	9/9
	Downslanting palpebral fissures	+	-	-	4/36	5/8
	Depressed nasal bridge	-	-	-	10/46	1/1
	Long upper lip	+	-	-	15/36	-
	Low-set ears	+	7/10	na	20/46	-
	Micrognathia	+	310	-	14/46	-
	**Abnormal dentition** (tooth discoloration, dysplastic teeth)	-	5/10	na	17/46	8/9
	Cleft palate	+	3/10	1/22	6/46	-
	Sparse scalp hair	-	5/8	na	4/36	-
	**Characteristic radiographic findings**					
	Platyspondyly	-	-	-	13/36	9/9
	Anterior beak of vertebral body	-	-	-	22/46	-
	Short ilium	-	-	-	27/46	-
	Prominent lesser trochanter	-	-	-	11/36	-
	Acetabular dysplasia	-	-	-	11/46	-
	Metaphyseal flaring	+	4/8	na	23/46	-
	Metaphyseal dysplasia of femoral head	-	-	na	19/46	2/2
	Elbow malalignment	+	-	-	10/36	-
	Radial head dislocation	-	5/10	12/21	15/36	-
	Overtubulation	-	-	na	6/36	-
	Bowing of long bones	+	6/10	-	13/46	-
	Generalized osteoporosis	-	na	-	12/36	-
	Healed fractures	-	1/10	na	21/46	na
	Radioulnar synostosis	+	8/10	10/21	1/36	na
	**Ascending aortic aneurysm**	-	-	na	2/36	na
	**Lung hypoplasia, restrictive lung disease**	-	-	na	3/36	na
***SLC39A13*-spEDS**	**Protuberant eyes with blue sclerae**	-	-	na	-	9/9
	**Hand with finely wrinkled palms**	-	-	na	10/36	9/9
	**Atrophy of the thenar muscles, and tapering fingers**	-	na	-	na	8/8
	**Hypermobility of distal joints**	+	na	-	37/46	7/9
	**Characteristic radiologic finding**					
	Moderate platyspondyly	-	-	-	16/36	9/9
	Mild to moderate osteopenia of the spine	+	6/10	-	12/36	7/9
	Small ileum	-	-	-	-	8/8
	Flat proximal femoral epiphyses	-	-	-	-	8/8
	Short, wide femoral necks	-	-	-	-	8/8
	**OTHER**					
	Scoliosis/kyphosis	+	2/10	6/22	32/36	1/1
	Hearing loss	-	2/10	-	2/46	-
	Easy bruising	+	na	na	3/10	8/9
	Atrophic scarring	+	4/32	-	10/46	5/7

B4GALT7-LRS, Larsen of Reunion Island syndrome patients with homozygous *B4GALT7* c.808C>T, p.(Arg270Cys) variant; spEDS, spondylodysplastic Ehlers-Danlos syndrome; na, not available, +, present; - absent.

Supplementary Table 3

Summary of ES results						
	**Million reads (% on target)**	**Mean coverage**	**% bases covered 20x**	**Exons fully covered**	**Variants (SNP/INDELs)**	**Filtered variants (SNP(INDELs)**
**Patient**	74,96 (98,7%)	62,35	85,01%	95,08%	115881/9998	43969/4418
**Father**	65,78 (98,2%)	55,78	82,79%	94,74%	114984/9737	43819/4443
**Mother**	65,96 (99,1%)	57,61	83,20%	95,13%	93326/5872	39130/3184

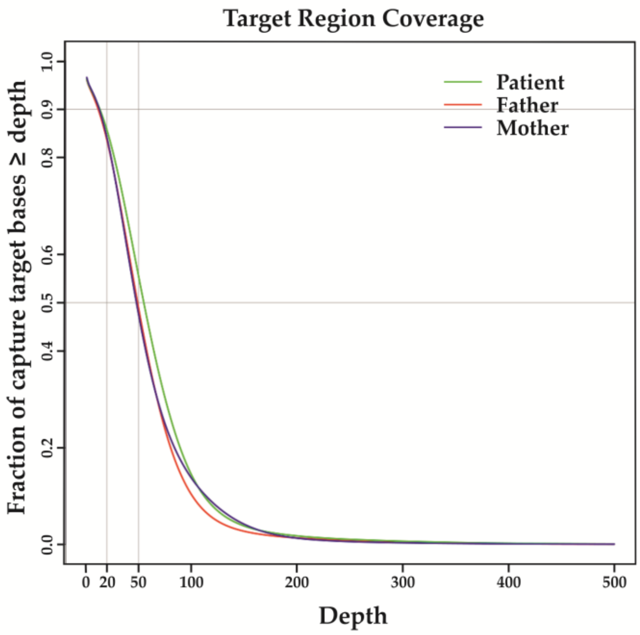

Supplementary Figure 1: Coverage distribution

Supplementary Table 4

Variants with perfect segregation among trio members			
**Pipeline**	**Total (DeNovo/CompHet/ Recessive)**	**SNPs (DeNovo/CompHet/ Recessive)**	**INDELs (DeNovo/CompHet/ Recessive)**
**Rare (MAF <0.01)**	3.280 / 4.800 / 766	2.765 / 4.440 / 560	515 / 893 / 206
**Functional variants** (missense, stop- or splice-affecting, CDS indels)	70 / 126 / 27	69 / 125 / 25	1 / 1 / 2
**Not in internal ES database**	68 / 110 / 25	67 / 109 / 23	1 / 1 / 2
**Deleterious:** DANN > 0.95 and M-CAP > 0.025	17 / 10 / 10	16 / 9 / 8	1 / 1 / 2
**Best in genes with:** RVIS < -0.24* and GDI<13.84	5 / 4 / 6	5 / 4 / 4	0 / 0 / 2
**Perfect segregation**	5 / 2 / 0	5 / 2 / 0	0 / 0 / 0

* average RVIS value suggested in PMID: 23990802 for connective tissue disorder
